# An Easy Method of Removing a Part of the Inferior Dental Nerve within the Lower Jaw

**Published:** 1875-08

**Authors:** John T. Hogden


### ARTICLE VIII.

### *An Easy Method of Removing a Part of the Inferior Dental Nerve within the Lower Jaws.*

BY JOHN T. HOGDEN, M. D.

In July, 1874, Mr. B., aged sixty two, of robust constitution, having had uniform good health, presenting himself, complaining of intense paroxysmal attacks of neuralgia. The site of pains was the gums, teeth and bone of the right inferior maxilla.

With a strong knife I made a cut one inch long, beginning on the inner side of the base of projecting ridge of the coronoid process, and running forward on the body of the bone to about the former site of the last molar tooth.

The point of the drill of a burring engine was entered just in front of the base of the coronoid process, and directed downward, backward and a little outward; in a few seconds the drill had penetrated the canal, as was known by the absence of resistance, the sudden twinge of pain and the flow of blood.

The small drill was then replaced by a globular burr one-eighth of an inch in diameter, with this the opening was enlarged until the nerve was again touched; the burr was now carried outward and inward, and then toward the posterior dental foramen; this last gave intense pain. Now the burr could be made to touch every part of the wall of the space without pain; the blood flowed pretty freely when the canal was first opened, and was still flowing—a little cotton pressed into the opening in the bone, the bleeding at once ceased, and we left the patient comfortable and happy.

For several days he suffered from nothing but soreness at the point of operation; two weeks later he called at my office, suffering a little with the old pain; this however, disappeared entirely, so that on the 21st of November, when we called on him to learn the finale, he was quite well and had much improved in flesh.—*London Monthly Review.*

---

